# Brain Metastasis in Triple-Negative Breast Cancer

**DOI:** 10.1155/2024/8816102

**Published:** 2024-09-30

**Authors:** Eduarda Bustamante, Fresia Casas, Renato Luque, Luis Piedra, Shamir Barros-Sevillano, Diego Chambergo-Michilot, J. Smith Torres-Roman, Alexis Narvaez-Rojas, Zaida Morante, Daniel Enriquez-Vera, Anshumi Desai, Cesar Razuri, Gabriel De la Cruz-Ku, Jhajaira Araujo

**Affiliations:** ^1^ Universidad Peruana de Ciencias Aplicadas, Lima, Peru; ^2^ Universidad Peruana Cayetano Heredia, Lima, Peru; ^3^ Universidad Científica Del Sur, Lima, Peru; ^4^ Facultad de Ciencias de la Salud Escuela de Medicina Universidad César Vallejo, Trujillo, Peru; ^5^ South American Center for Education and Research Public Health Universidad Norbert Wiener, Lima, Peru; ^6^ University of Miami Miller School of Medicine, Miami, FL, USA; ^7^ Instituto Nacional de Enfermedades Neoplasicas, Lima, Peru; ^8^ Universidad Ricardo Palma, Lima, Peru; ^9^ Escuela Profesional de Medicina Humana Universidad Privada San Juan Bautista, Chorrillos, Lima, Peru; ^10^ Centro de Investigación Básica y Traslacional Auna-Ideas, Lima, Peru

## Abstract

**Background:**

Breast cancer is an important cause of cancer-related death in women worldwide and represents the second most frequent cause of brain metastases after lung cancer. The aim of this study was to determine the characteristics and outcomes of triple-negative breast cancer (TNBC) patients with brain metastasis (BM).

**Methods:**

We retrospectively reviewed a cohort of patients diagnosed with TNBC at the “Instituto Nacional de Enfermedades Neoplasicas” (period 2000–2014) to evaluate patients who developed BM. Survival rates were assessed by the Kaplan–Meier method, and prognostic factors were identified with the Cox regression analysis.

**Results:**

Of a total of 2007 TNBC patients, 193 (9.62%) developed BM. Of these, 169 stages I–III patients with a median age of 45 years (range:21–78) were included. The stage in this cohort was 4 (2.4%) clinical stage (CS) I, 23 (13.6%) with CS II and 142 (84.0%) with CS III. Most of these patients presented ECOG ≥2 (68.6%). The most common symptom was headache (74.0%), followed by nausea-vomiting (46.7%). Imaging showed that 80 patients (53.0%) had ≥1 metastatic brain lesion. Regarding the treatment of BM in this cohort, 132 patients (84.6%) received radiotherapy (RT), 2 (1.5%) surgery, and 6 (4.5%) surgery plus RT. The overall survival (OS) rate of BM was 59.8%, 37.3%, and 15.0% at 3, 6, and 12 months, respectively. A multivariate analysis showed RT to be the only factor with a positive impact on the OS of BM (hazard ratio (HR) = 0.48, 95% confidence interval (CI):0.30-0.77, and *p* = 0.002), while ECOG ≥2 was associated with a worse OS (HR = 1.69, 95%CI:1.15–2.48, and *p* = 0.007).

**Conclusion:**

Despite the poor prognosis of TNBC patients who develop BM, RT showed a benefit in OS rates, while ECOG ≥2 was the only prognostic factor associated with a worse OS. These results may be useful for multidisciplinary teams for treatment planning in patients with TNBC and BM.

## 1. Introduction

Breast cancer (BC) is the most prevalent malignancy and represents the leading cause of cancer-related death among women worldwide [1]. Despite this, improvements in systemic and targeted therapies have increased the overall survival (OS) in women with BC. There are four molecular subtypes of BC [2, 3]. Of these, triple-negative breast cancer (TNBC) is characterized by its aggressiveness and poor long-term survival compared to the luminal subtypes [4, 5]. TNBC has a tendency to develop visceral metastasis predominantly in the lungs, liver, and brain [6], and it is estimated that about 25% to 46% of women with TNBC will develop brain metastasis (BM) during the course of their disease [7–12].

Surgical and radiation therapies are often required in patients with TNBC with BM. The standard of care in patients with a low disease burden, good performance status, or significant mass effect is surgery followed by stereotactic brain radiotherapy. On the other hand, women with a poor performance status and multiple BM are usually offered whole-brain radiation therapy or best supportive care [13–15].

Previous studies have reported prognostic factors associated with a worse OS, which include the first recurrence occurring in the brain, more than three brain lesions, no BM-directed treatment, subsequent recurrent BM, symptomatic BM, and uncontrolled extracranial metastasis [10]. However, these studies mostly included Asian or North American patients. In the Latin American population, TNBC is has a high prevalence of 18% to 35% [16–18]. Thus, we aimed to determine the characteristics, outcomes, and prognostic factors among Latin American patients with TNBC and BM treated in a tertiary center.

## 2. Methods

### 2.1. Study Design and Patients

This was a retrospective single-center cohort study performed in the “Instituto Nacional de Enfermedades Neoplasticas” (INEN) which is a tertiary center national oncologic reference institute attending patients from all the regions in Peru.

The patients included in the study were diagnosed with TNBC from 2000 to 2014. The follow-up was until March, 2021. The data were collected from December 2017 to December, 2022. All the patients had pathologic confirmation of BC. Diagnosis of TNBC was based on immunohistochemistry (IHC) with estrogen receptor (ER) negative (IHC <1%), progesterone receptor negative (IHC <1%), and human epidermal growth factor receptor 2 (HER2) negative. In cases with inconclusive results for HER2, fluorescence *in situ* hybridization was performed to confirm the negativity. Staging of the patients was done according to the 8th edition of the American Joint Committee on Cancer (AJCC) [19].

Women diagnosed with TNBC who developed BM independently of the clinical stage (CS) and treatment received during the study period were included in the study. Patients younger than 18 years old, with clinical stage IV or unknown clinical stage, and patients lost to follow-up were excluded. The study sample included all patients who met the inclusion and exclusion criteria. In regard to the diagnosis and management of the central nervous system metastasis, this is not part of the initial screening in patients with locally advanced breast cancer but rather is only performed in patients with neurologic symptoms at the diagnosis or during treatment and follow-up. The protocol for locally advanced TNBC of our institution is neoadjuvant chemotherapy (doxorubicin, cyclophosphamide, and taxanes), followed by surgery (more than 65% being mastectomies), and complementary radiotherapy. However, one third of the patients were referred after initial surgery without neoadjuvant chemotherapy and received adjuvant chemotherapy. The use of radiotherapy after mastectomy remains controversial; however, clear consensus followed by the National Comprehensive Cancer Network (NCCN) guidelines recommend radiotherapy in patients with more than four positive nodes after mastectomy or in those with high risk features [19]. Moreover, in our center, in cases presenting disease progression during active treatment, radiotherapy is also carried out and freely covered as part of the treatment according to the “Plan Esperanza” implemented in 2012. Finally, patients with a lower burden of disease including CS II or less than four positive nodes who underwent mastectomy were not considered candidates for complementary radiotherapy.

### 2.2. Follow-Up

We defined OS of BM as the time from the diagnosis of BM until the date of death or end of the study. All the metastases were diagnosed by computed tomography or magnetic resonance imaging.

### 2.3. Statistical Analysis

The distribution of continuous variables was evaluated with the Kolmogorov–Smirnov test, and medians and range were estimated. For qualitative variables, these were expressed as frequencies and percentages. Moreover, the Kaplan–Meier method was used to obtain the survival curves, while the log-rank test estimated differences between groups. To identify the prognostic factors, the Cox regression model was used for the univariate and multivariate analyses. We performed a sensitivity analysis which included only patients who received whole-brain radiation. In order to address any potential sources of bias, all the population available who met our inclusion and exclusion criteria was included in our analyses. A *p* value <0.05 was considered statistically significant. The data were analyzed using the Statistical Package for the Social Sciences (SPSS) software v26.0 and STATA v17.

### 2.4. Ethical Aspects

This study was approved by the Institutional Research Ethics of the “Instituto Nacional de Enfermedades Neoplásicas” (IRB No: INEN 16–46). All the information was maintained in a confidential manner, removing all personal information related to patients or physicians. The data were used exclusively for this study and were not shared with any other parties. The need for informed consent was waived by the Ethics Committee due to the retrospective study design. All the identities of the patients were blinded, replacing the information with unrelated codes. During data collection, the authors had access to information that could identify individual participants. After data collection, authors did not have access to individual patient information. All procedures and treatments performed were in accordance with the ethical standards of the Institutional and National Research Committee and with the 1964 Declaration of Helsinki and its later amendments.

## 3. Results

### 3.1. Sociodemographic and Clinical Characteristics

From a total of 2007 TNBC patients, 169 patients (8.4%) who developed BM and were CS I–III were included in the analysis. In this cohort, the median age was 45 years (range: 21–78 years), 57.4% were premenopausal and 11.2% had a family history of breast and/or ovary cancer. Moreover, the majority of the population was diagnosed with CS III 142 (84.0%) (Table 1).

### 3.2. Primary Management

Of these 169 patients, surgery was performed in 136 (80.5%) patients, with the majority undergoing mastectomy (65.7%, *N* = 111). Regarding chemotherapy, 114 patients received neoadjuvant chemotherapy (67.5%), while 70 received adjuvant chemotherapy (41.4%). Local radiotherapy was administered to 105 patients (62.1%) (Table 1).

### 3.3. Relapse in Patients with Triple-Negative Breast Cancer and Brain Metastasis

The median time from the diagnosis to disease progression to the brain was 18.0 months (range: 2–185 months). In addition to the brain, the metastasis to other organs in this cohort was 55.6% (*N* = 94); the most frequent organs being the lung (39.1%), followed by bone (20.7%), and liver (16.0%). In addition, 27.2% of patients had locoregional relapse (Table 2).

### 3.4. Brain Metastasis and Treatment

Most of patients in this cohort of 169 patients presented symptoms of BM (*N* = 153; 90.5%). The median time from symptom onset to the diagnosis of BM was 7 days (range: 1–60 days). The most common symptom was headache (74.0%), followed by nausea and vomiting (46.7%), ataxia (13.0%), muscle weakness (11.2%), and seizures (5.3%). Furthermore, 68.6% of patients presented with an Eastern Cooperative Oncology Group (ECOG) score ≥2. In patients in whom the number of lesions was reported (*N* = 151), the majority had more than one BM (53.0%) (Table 3).

Among the patients who received treatment, 126 (94.0%) received only radiotherapy, whereas two (1.5%) only underwent surgical resection. Moreover, six patients underwent surgical resection followed by radiotherapy (4.5%). Patients who underwent radiotherapy (*N* = 132) received a median dose of 3000 cGy and 12 patients (9.1%) received <3000 cGy (Table 3).

## 4. Survival Outcomes

**Figure 1:**
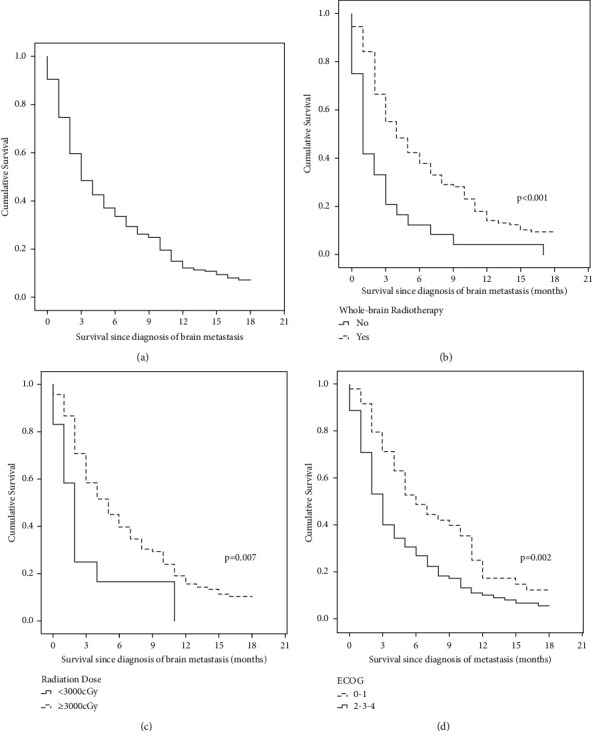
Survival following the diagnosis of brain metastasis in patients with triple-negative breast cancer: (a) overall population; (b) according to the ECOG; (c) according to the treatment with radiotherapy; (d) according to radiotherapy doses.

The median follow-up was 45 months. The OS of BM in our cohort with stages I–III was 59.8%, 37.3%, and 15.0% at 3, 6, and 12 months, respectively (Table 4). Patients with ECOG 0–1 and those who received radiotherapy and a radiotherapy dose ≥3000 cGy presented a better OS of BM (Figure 1 and Table 4).

In the univariate analysis, radiotherapy was associated with a better OS of BM, while an ECOG ≥2 and the presence of more than one lesion were correlated with a worse OS. In the multivariate analysis, radiotherapy was the only factor showing a positive impact on OS in patients with BM (hazard ratio (HR) = 0.48, 95% confidence interval (CI): 0.30–0.77, and *p* = 0.002), and an ECOG ≥2 was associated with a worse OS (HR: 1.69, 95%CI: 1.15–2.48, and *p* = 0.007) (Tables 5 and 6).

## 5. Discussion

In this study, we describe the characteristics and outcomes of 169 Latin American patients diagnosed with TNBC CS I–III, who developed BM. The majority were diagnosed at CS III, and the most common organ involved in addition to the brain was the lung. Radiotherapy was the main treatment used for BM and was found to be a prognostic factor of a better OS.

Regarding the clinical characteristics of the patients, the mean age of our patients was 45 years, being similar to a study conducted in Taiwan [20], in which the median age at the diagnosis of TNBC was 49 years, and another study at the Fudan University Shanghai Cancer Center [10], in which the median age at the diagnosis was 48 years.

A systematic review by Koniali et al. identified young age, ER negativity, HER-2 receptor positivity, high tumor stage, size, histological grade, high Ki67 labeling index, and nodal involvement as independent risk factors for BM in patients with BC [21]. In addition, other studies suggest that positive lymph nodes, grade 3, higher stage, TNBC, and HER2 positivity are risk factors for BM [10, 22]. Although we did not determine the risk factors for BM, we found that most of our population was diagnosed at an age younger than 50 years, with an advanced stage, T4, histological grade III, and had node involvement. The frequency of these characteristics is also consistent with previous reports in Hispanic patients. [16, 23].

We found that 43.8% of metastatic TNBC patients diagnosed with BM as the first site of relapse were diagnosed with extracranial metastasis at the same time. The most common sites involved were the lymph nodes followed by the lung, bone, and liver [10]. We included TNBC patients with CS I–III and found that 55.6% developed metastases to other organs. The most common metastatic extracranial organs were the lung, then bone, and finally, the liver. Compared to other BC subtypes, the metastasis in bones and the liver are less common in TNBC patients with BM, while metastasis in the lung is more frequent [11].

In relation to symptoms, our results showed that most of patients presented headache as the initial symptom. Similarly, previous studies have reported neurological symptoms as the most frequent, including headache followed by vomiting and motor involvement [10]. We also found that 9.5% of patients did not present any symptoms. Despite the NCCN and American Society of Clinical Oncology guidelines not recommending screening for asymptomatic BM, a recent study suggested that screening of asymptomatic BM in metastatic TNBC should be evaluated [9].

The OS after the diagnosis of BM in the present study was 3 months, which is shorter than the median survival of 7.3 months in a study conducted in China that included only metastatic TNBC [10] but was similar to another study conducted in Turkey (3.5 months) [24]. Poorer survival outcomes have been reported in Latin America compared to Europe, Asia, or the United States, which may be partly explained by disparities in access to treatment, late diagnosis, and the genetic characteristics of this population [25–27].

In regards to the prognostic factors of OS in patients with BM, good performance status and radiotherapy were significantly associated with improved survival, confirming the findings of previous studies [28, 29]. Patients who received radiotherapy had a reduction of 0.62 in the HR, whilst in those receiving a dose ≥3000 cGy, the risk decreased to 0.64. Due to the limited number of patients that underwent surgery plus radiotherapy or surgery alone, we could not compare survival according to the different treatment modalities. However, it has been reported that surgery plus radiotherapy improves OS and the control of BM symptoms versus radiation therapy alone [30, 31], and in patients with only one lesion, this treatment reduces the rate of recurrence and death due to neurologic causes [32].

Whole brain radiation therapy (WBRT) has been the main modality of treatment for years due to the achievement of better survival [33]; however, the results of two clinical trials have shown that postoperative stereotactic radiosurgery (SRS) presents the same results in OS but with a lower risk of cognitive impairment [34, 35]. Furthermore, when pre-versus post-SRS were compared, recurrence and OS were similar but symptomatic radiation necrosis and leptomeningeal disease were lower in the pre-SRS group [36]. SRS is also recommended over WBRT when surgery is not possible due to the reduction in the risk of neurocognitive dysfunction but with no differences in progression and survival outcomes [37, 38].

Despite the germline BRCA mutation information being not available in our study, it is important to consider that the recent literature has shown that this special group of TNBC patients with gBRCA1 has higher rates of brain metastasis [39]. Moreover, these patients have a shorter interval to brain progression and worse survival after the diagnosis of CNS disease compared to noncarriers [40, 41]. Therefore, further studies that assess the efficacy of the new antibody-drug conjugates and checkpoint inhibitors are needed for noncarriers and gBRCA1/2 TNBC patients with BM [42].

## 6. Limitations

Our study has some limitations. First, the study is retrospective; therefore, we could only determine association but not causation. Second, although we describe the characteristics and outcomes of TNBC with BM in a Latin American population, our population may not be representative of the general Latin American population since some records were incomplete and only one reference institute was included. Third, although we evaluated a Latin American population and our results might serve for discussion of the prognosis among patients with TNBC, only Peruvian patients were included in the study. Therefore, an international multicentric study would be necessary to allow generalization of the results.

## 7. Conclusions

In conclusion, despite the poor prognosis of TNBC patients who develop BM, radiotherapy showed a better OS rate of 49%, while an ECOG score ≥2 was the only prognostic factor associated with a worse OS. These results may be useful for multidisciplinary teams for treatment planning in TNBC patients with BM. However, new prospective studies are needed to define prognostic factors among TNBC patients with BM. Radiotherapy improves survival, while ECOG can be used as a predictor of a poor prognosis.

## Figures and Tables

**Table 1:** Sociodemographic, clinical characteristics, and primary treatment of patients with triple-negative breast cancer and brain metastases.

	Frequency (N)	Percentage (%)
Patients	169	100

*Age*		
Median (range)	45 (21–78)	
<60	146	86.4
≥60	23	13.6

*BMI*		
Normal	44	31.9
Overweight	54	39.1
Obesity type 1	31	22.5
Obesity type 2	8	5.8
Obesity type 3	1	0.7
Unknown	31	

*Menopausal status*		
Premenopausal	97	57.4
Postmenopausal	69	40.8
Unknown	3	

*FHBOC*		
No	150	88.8
Yes	19	11.2

*Laterality*		
Right	79	46.7
Left	84	49.7
Bilateral	6	3.6

*Histological grade*		
II	37	21.9
III	115	68.0
Unknown	17	

*T Stage*		
T1	2	1.2
T2	37	21.9
T3	35	20.7
T4	90	53.3
Unknown	5	

*N*		
N0	36	21.3
N1	71	42.0
N2	39	23.1
N3	19	11.2
Unknown	4	

*AJCC staging*		
I	4	2.4
II	23	13.6
III	142	84.0

*Neoadjuvant chemotherapy for primary cancer*		
No	55	32.5
Yes	114	67.5

*Surgery*		
No	33	19.5
Yes	136	80.5

*Type of surgery (N* *=* *136)*		
Conservative	25	14.8
Mastectomy	111	65.7

*Local radiotherapy*		
No	64	37.9
Yes	105	62.1

*Adjuvant chemotherapy*		
No	99	58.6
Yes	70	41.4

BMI, body mass index; FHBOC, family history of breast and/or ovarian cancer, AJCC, American Joint Committee on Cancer.

**Table 2:** Relapse patterns and organs involved in patients with triple-negative breast cancer and brain metastases.

	Frequency (N)	Percentage (%)
Patients	169	100

*Locoregional relapse*		
No	123	72.8
Yes	46	27.2
Time from diagnosis to brain metastasis (months) median (range)	18.0 (2–185)	

*Liver*		
No	142	84.0
Yes	27	16.0

*Lung*		
No	103	60.9
Yes	66	39.1

*Bone*		
No	134	79.3
Yes	35	20.7

*Number of other organs involved*		
0	75	44.4
1	57	33.7
2	31	18.3
3	4	2.4
4	2	1.2

**Table 3:** Clinical and pathological characteristics of brain metastasis in patients with triple-negative breast cancer.

	Frequency (N)	Percentage (%)
Patients	169	100
Time from onset of symptoms to diagnosis (days) median (range)	7.0 (1–60)	

*Symptoms*		
Headache	125	74.0
Nausea-vomiting	79	46.7
Ataxia	22	13.0
Muscle weakness	19	11.2
Seizures	9	5.3

*ECOG*		
0–1	49	31.4
≥2	107	68.6
Unknown	13	

*Number of brain lesions*		
1	71	47.0
≥1	80	53.0
Unknown	18	

*Treatment*		
No	20	12.8%
Yes	136	87.2%
Unknown	13	

*Treatment type (N* *=* *136)*		
Surgery	2	1.5
Surgery + RT	6	4.5
Only RT	126	94.0
Unknown	2	

*RT*		
No	24	15.4
Yes	132	84.6
Unknown	13	
RT dose (cGy) (*N* = 132) median (range)	3000 (2000–3900)	
<3000 cGy	12	9.1
≥3000 cGy	120	90.9

RT: radiotherapy; ECOG: Eastern Cooperative Oncology Group.

**Table 4:** Survival following the diagnosis of brain metastasis in patients with triple-negative breast cancer.

Survival outcomes	Total population	Median – months (95% CI)	Time periods					
			1 month (%)	2 months (%)	3 months (%)	6 months (%)	12 months (%)	*p* Value
Overall survival	169	3 (2.1–3.9)	90.5	74.6	59.8	37.3	15.0	—

*AJCC staging*								
I	4	2 (0.3–3.7)	100	25.0	25.0	25.0	0.0	0.584
II	23	3 (1.4–4.6)	87.0	69.6	47.8	39.1	10.4	
III	142	3 (1.9–4.0)	72.5	59.2	49.3	33.0	12.9	

*Radiotherapy*								
No	24	1 (0.4–1.6)	75.0	41.7	33.3	12.5	4.2	<0.001
Yes	132	4 (2.7–5.3)	94.7	84.1	66.7	42.4	17.8	

*RT doses (cGy)*								
<3000 cGy	12	2 (1.3–2.7)	83.3	58.3	25.0	16.7	0.0	0.007
≥3000 cGy	120	5 (3.7–6.3)	95.8	86.7	70.8	45.0	19.2	

*ECOG*								
0–1	49	6 (3.7–8.3)	98.0	91.8	79.6	53.1	25.3	0.001
≥2	107	3 (2.4–3.6)	88.8	71.0	53.3	30.8	10.3	

AJCC: American Joint Committee on Cancer; CI: confidence interval; RT: radiotherapy; ECOG: Eastern Cooperative Oncology Group.

**Table 5:** Univariate and multivariate Cox regression analysis in patients with triple-negative breast cancer who developed brain metastases.

Characteristics	Survival following the diagnosis of metastasis						
	No. of patients (*N* = 169)	Univariate analysis			Multivariate analysis		
		HR	95% CI	*p* Value	HR	95% CI	*p* value
Age	169	1.01	0.99–1.02	0.433	1.01	0.99–1.02	0.262

*FHBOC*							
No	150	1.00			1.00		
Yes	19	0.78	0.45–1.32	0.350	0.81	0.44–1.49	0.498

*ECOG*							
0–1	49	1.00			1.00		
≥2	107	1.78	1.23–2.56	0.002	1.69	1.15–2.48	0.007

*AJCC staging*							
I–II	27	1.00			1.00		
III	142	0.98	0.64–1.51	0.929	1.14	0.71–1.84	0.584

*Lesions*							
1	71	1.00			1.00		
≥1	80	1.52	1.08–2.14	0.016	1.35	0.95–1.91	0.092

*Radiotherapy*							
No	24	1.00			1.00		
Yes	132	0.43	0.27–0.67	<0.001	0.48	0.30–0.77	0.002
Number of other organs involved	169	1.11	0.94–1.32	0.219	1.15	0.95–1.40	0.165

FHBOC: family history of breast and/or ovarian cancer; AJCC: American Joint Committee on Cancer; ECOG: Eastern Cooperative Oncology Group; HR: hazard ratio; CI: confidence interval. Subgroup analysis of patients who received radiotherapy, an ECOG ≥2 (HR: 1.96, 95% CI: 1.29–2.97, *p* = 0.002) and radiotherapy with ≥3000 cGy (HR: 0.36, 95% CI: 0.18–0.74, *p* = 0.005) were significant prognostic factors of OS (Table 6).

**Table 6:** Univariate and multivariate Cox regression analysis in patients with triple-negative breast cancer who developed brain metastases and received radiotherapy.

Characteristics	Survival following the diagnosis of metastasis						
	No. of patients (*N* = 132)	Univariate analysis			Multivariate analysis		
		HR	95% CI	*p* Value	HR	95% CI	*p* value
Age	132	1.00	0.99–1.02	0.937	1.00	0.98–1.02	0.829

*FHBOC*							
No	119	1.00			1.00		
Yes	13	0.53	0.27–1.06	0.072	0.68	0.34–1.37	0.283

*ECOG*							
0–1	45	1.00			1.00		
≥2	87	1.72	1.16–2.53	0.007	1.96	1.29–2.97	0.002

*AJCC staging*							
I-II	21	1.00			1.00		
III	111	0.94	0.58–1.54	0.811	1.02	0.61–1.71	0.945

*Lesions*							
1	63	1.00			1.00		
≥1	65	1.55	1.07–2.25	0.021	1.51	1.03–2.21	0.033

*Radiotherapy*							
<3000 cGy	12	1.00			1.00		
≥3000 cGy	120	0.46	0.24–0.86	0.014	0.36	0.18–0.74	0.005
Number of other organs involved	132	1.14	0.94–1.39	0.176	1.07	0.86–1.33	0.569

FHBOC: family history of breast and/or ovarian cancer; AJCC: American Joint Committee on Cancer; ECOG: Eastern Cooperative Oncology Group; HR: hazard ratio; CI: confidence interval.

## Data Availability

Deidentified data can be shared upon reasonable request to the corresponding author.
